# Alternative RNA Structure-Coupled Gene Regulations in Tumorigenesis

**DOI:** 10.3390/ijms16010452

**Published:** 2014-12-29

**Authors:** Feng-Chi Chen

**Affiliations:** 1 Institute of Population Health Sciences, National Health Research Institutes, Miaoli County 350, Taiwan; E-Mail: fcchen@nrhi.org.tw; Tel.: +886-37-246-166 (ext. 36111); Fax: +886-37-586-467; 2 Department of Biological Science and Technology, National Chiao-Tung University, Hsinchu 300, Taiwan; 3 Department of Dentistry, China Medical University, Taichung 404, Taiwan

**Keywords:** gene regulation, alternative splicing, alternative promoter usage, alternative cleavage and polyadenylation, untranslated region, nonsense-mediated decay, upstream open reading frame, internal ribosome entry site, microRNA, tumorigenesis

## Abstract

Alternative RNA structures (ARSs), or alternative transcript isoforms, are critical for regulating cellular phenotypes in humans. In addition to generating functionally diverse protein isoforms from a single gene, ARS can alter the sequence contents of 5'/3' untranslated regions (UTRs) and intronic regions, thus also affecting the regulatory effects of these regions. ARS may introduce premature stop codon(s) into a transcript, and render the transcript susceptible to nonsense-mediated decay, which in turn can influence the overall gene expression level. Meanwhile, ARS can regulate the presence/absence of upstream open reading frames and microRNA targeting sites in 5'UTRs and 3'UTRs, respectively, thus affecting translational efficiencies and protein expression levels. Furthermore, since ARS may alter exon-intron structures, it can influence the biogenesis of intronic microRNAs and indirectly affect the expression of the target genes of these microRNAs. The connections between ARS and multiple regulatory mechanisms underline the importance of ARS in determining cell fate. Accumulating evidence indicates that ARS-coupled regulations play important roles in tumorigenesis. Here I will review our current knowledge in this field, and discuss potential future directions.

## 1. Introduction

The regulatory sophistication of human genes is largely ascribable to the complexity of the transcriptome. A single gene can be transcribed into multiple transcript isoforms, or alternative RNA structures (ARSs). There are three major mechanisms for the generation of ARSs: alternative RNA splicing (AS), alternative promoter selection (AP), and alternative cleavage and polyadenylation (AC). AS can alter the compositions of 5' untranslated regions (5'UTRs), coding sequences (CDSs), and 3'UTRs. Meanwhile, AP and AC affect mostly 5'UTRs and 3'UTRs, respectively. Each of these three post-transcriptional mechanisms can profoundly affect cellular phenotypes. Since AS is much more prevalent than AP and AC in human cells, here I will focus mainly on the regulatory roles of AS in tumorigenesis, and extend the discussions to the other two mechanisms when appropriate.

AS plays a central role in gene regulation in complex organisms. In humans, AS occurs in more than 95% of multi-exonic coding genes [1] and a large proportion of noncoding genes [2]. AS has been shown to participate in a wide range of biological processes, including metabolism [3,4], differentiation [5,6], pluripotency [7,8,9], adhesion [10,11], cell proliferation [12,13], and apoptosis [14,15], to name but a few. With such prevalence and biological versatility in human cells, AS is understandably involved in the pathogenesis of many human diseases [16,17,18,19], including various types of cancers [20,21,22]. One well-established connection between AS and cancer is the oncogenic transcript/protein isoforms generated due to dysregulated splicing, which can skew regulatory/signal pathways towards tumorigenesis [23,24,25]. In addition to its influences on protein isoforms, AS has also recently been found to be widely involved in the gene regulatory networks of cancer cells [23,24,25].

Although AS is traditionally considered as a post-transcriptional regulatory mechanism, recent evidence indicated that splicing actually occurred co-transcriptionally in most cases [26]. Therefore, AS and transcription have been proposed to be co-regulated [26]. In other words, interferences in the transcriptional process may alter the splicing pattern of a gene, and vice versa. This intriguing phenomenon of co-regulation highlights an underappreciated role of RNA splicing in the gene regulatory network. In addition to the transcriptional process, AS is also closely related to other gene regulatory mechanisms. Importantly, AS can change the composition of the untranslated regions (UTRs) and/or the coding sequences (CDS) of transcripts. To be sure, 5' and 3'UTR sequences can also be changed because of AP and AC, respectively. These two regulatory mechanisms may occur with or without the concurrence of AS.

**Figure 1:**
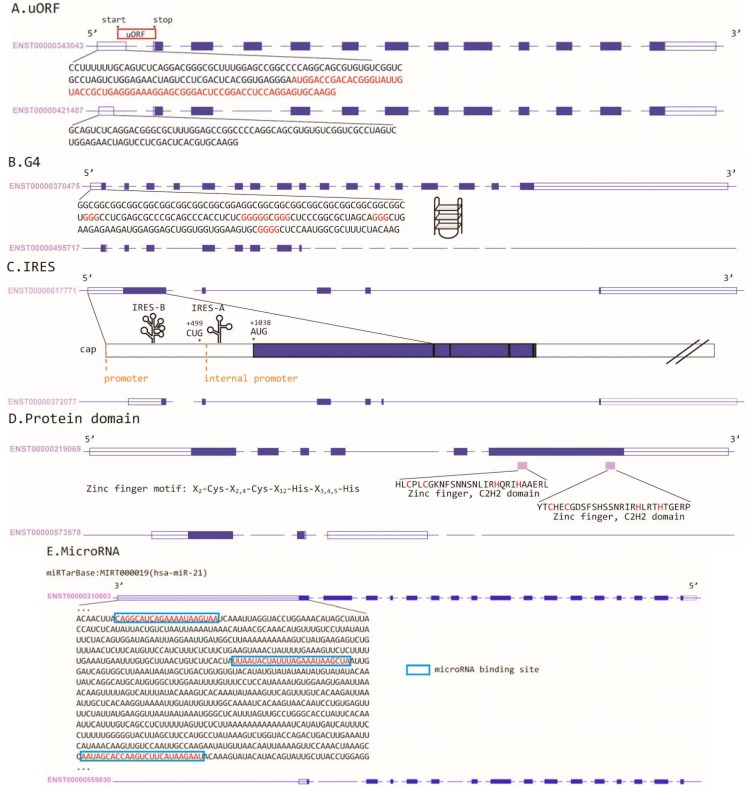
Alternative RNA structure-regulated presence/absence of (**A**) upstream open reading frame (uORF); (**B**) G-quadruplex (G4); (**C**) internal ribosomal entry sites (IRES); (**D**) protein domain (zinc finger domain in this example); and (**E**) microRNA binding site. The empty boxes and the blue boxes, respectively, represent UTR exons and coding exons.

Figure 1 demonstrates how ARS can affect the presence/absence of regulatory motifs. An alternative 5'UTR may by chance include an AUG translational start codon (Figure 1A). According to the scanning model of protein translation, the translation machinery starts scanning from the 5'cap of an mRNA and initiates translation at the first encountered AUG given adequate sequence contexts [27]. In Figure 1A, an AUG is located upstream of the canonical start codon of the downstream CDS (the beginning of the blue boxes). The upstream open reading frame (uORF) starting from the AUG extends to the interior of the first coding exon, and has a different reading frame from that of the downstream main CDS. This uORF thus may “hijack” the translational machinery, causing skipping of the canonical start codon and translational inhibition. If a 5'UTR contains multiple runs of ≥3 guanines, as is shown in Figure 1B, these guanine runs may fold into a steric G-quadruplex (G4) structure, leading to stalling of the scanning ribosome and translational inhibition.

Alternative 5'UTRs may also introduce alternative internal ribosomal entry sites (IRESs; Figure 1C), which can mediate cap-independent protein translation and regulate protein translation. Meanwhile, transcript isoforms usually are translated into peptides of different lengths. Particularly, alternative coding exons may contain important protein domains (Figure 1D), thus affecting the interactions between a protein and other macromolecules (DNA, RNA, or protein). 3'UTRs of different lengths can be differentially targeted by microRNAs. Figure 1E shows an example of how alternative 3'UTRs result in the presence/absence of microRNA binding sites.

**Figure 2:**
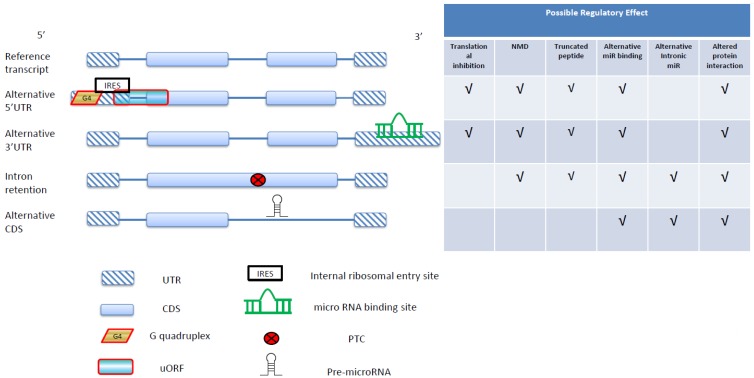
An overview of alternative RNA structure-coupled gene regulations. Four possible types of alternative RNA structure are listed (alternative 5'UTR, alternative 3'UTR, intron retention, and alternative CDS). The possible regulatory outcomes of the alternative RNA structures are shown in the table on the right side.

The inset table in Figure 2 summarizes the potential regulatory effects of ARS. In alternative 5'UTR, the presence/absence of translationally repressive uORFs or G4s could significantly affect the translational efficiency of the downstream main CDS [28,29]. uORFs, which by definition include a stop codon, can also induce nonsense-mediated decay (NMD) [30]. Alternative 5'UTR by itself can also result in alternative microRNA binding in these untranslated regions. Meanwhile, IRESs in alternative 5'UTRs may mediate protein translation to yield N-truncated peptides. This shortening of peptides may in turn affect protein–protein interactions or protein–DNA/RNA interactions. Alternative 3'UTRs can cause differential binding of microRNAs, leading to changes in the level of RNA/protein expression [31]. NMD may also be activated given long 3'UTRs [30]. In CDS, AS might lead to the production of truncated proteins by introducing premature termination codons (PTCs), which are frequently observed in the case of intron retention [32]. PTCs usually also induce NMD, and consequently a reduced level of RNA/protein expression [33,34]. Meanwhile, intron retention may also result in peptides of extended lengths, potentially leading to changes in protein interactions. Finally, ARS can result in a repertoire of functionally divergent proteins from a single gene, thus providing significant functional flexibility and a “switch” for spatiotemporal regulations [35].

With such a multiplicity of biological roles, ARS is inferred to take a central position in a multi-dimensional regulatory network that integrates epigenome, regulome, transcriptome, proteome, and interactome [36]. Dysregulated ARS could severely disrupt the regulatory network and, consequently, cellular phenotypes. Accordingly, ARS understandably plays an important role in the development of tumors [20,21,22]. Particularly, AS has been shown to contribute significantly to all of the hallmarks of cancer, including anti-apoptosis [37], angiogenesis [38,39], evasion from immune surveillance [40,41], metastasis [42], abnormal metabolism [43,44], and other cancer cell phenotypes. In the following I will review how ARS (especially AS) is coupled with other regulatory disruptions that might eventually lead to the development of cancers.

## 2. NMD Regulation in Cancer

AS can introduce reading frame shifts (and therefore PTCs) downstream of the alternative coding exonic regions when the lengths of such regions are not multiples of three. The resulting PCT-containing transcripts are usually targeted by NMD, thus affecting the overall level of gene expression [45,46].

**Figure 3:**
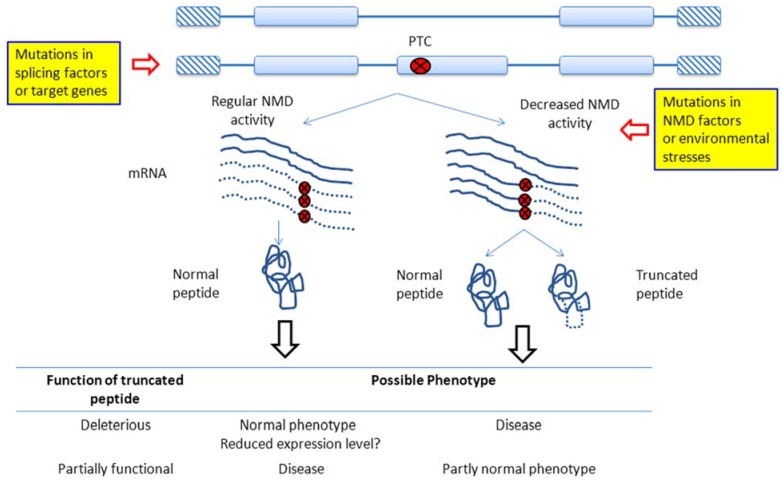
Possible outcomes of AS-NMD dysregulation. Misregulations of AS-NMD may occur because of mutations in the splicing factors or the genes of interest, which can lead to changes in the proportion of NMD-sensitive transcripts and subsequently the overall gene expression level (**left** pathway). Alternatively, aberrant AS-NMD can occur because of mutations in the NMD regulators. The resulting decreases in NMD activity cause truncated peptides to accumulate in the cell (**right** pathway). Generally, truncated peptides are detrimental and potentially pathogenic. However, in cases where truncated peptides are partially functional, decreases in NMD activity may turn out to be beneficial (**bottom** panel). The dashed curves indicate that the mRNA sequences are not translated.

Figure 3 shows possible reasons for NMD dysregulations and the phenotypic consequences. NMD dysregulation may result from mutations in splicing factors or the genes of interest (the left pathway in Figure 3), both of which can result in the occurrences of PTCs and thus altered ratios in NMD-sensible transcript isoforms. Alternatively, the dysregulation can be induced by mutations in NMD regulators, which usually lead to decreased NMD activities and the accumulation of truncated peptides (the right pathway in Figure 3). Of note, the phenotypic effects of NMD dysregulations depend on whether the truncated peptides are deleterious or partially functional (inset table at the bottom of Figure 3). If the truncated peptides are detrimental, the left pathway may yield normal phenotypes, whereas the right pathway can cause diseased phenotypes. Notably, however, in the left pathway, normal NMD activities may significantly reduce the overall gene expression level. In this case, the phenotypic effects will depend on the functional contexts of the affected genes, and no simple predictions can be made. Meanwhile, if the truncated peptides are partially functional, elimination (left pathway) and retention (right pathway) of these peptides may lead to diseased and partially normal phenotypes, respectively (Figure 3).

Interestingly, AS-NMD coupling also occurs in the autoregulation of splicing factors [47,48]. Jangi and colleagues [47] showed that Rbfox2, a key regulator of AS, could affect >200 AS-NMD events in mouse embryonic stem cells. About 70 of these events occurred to RNA-binding proteins (RBPs), many of which are splicing regulators. The authors suggested that the AS-NMD events could dampen the expression levels of RBP genes. Furthermore, by enhancing or repressing the NMD activities, the expression levels of different sets of splicing factors could be altered, leading to shifts in the splicing network [47].

Despite its effects on the overall level of gene expression, AS-NMD regulation does not necessarily yield observable changes in the relative abundance of transcript isoforms [47], possibly because of the short half-lives of the NMD-targeted transcripts. NMD is a double-edged sword in its effects on human health. On the one hand, it prevents the cell from producing truncated peptides, which are detrimental in most cases. On the other hand, the depletion of truncated peptides by NMD can be harmful if these peptides are partially functional (Figure 3). One good example is Ullrich disease. The collagen VI α2 gene in Ullrich disease patients contains a PTC. Yet the truncated collagen peptides are partially functional. It has been shown that inhibition of the NMD activity can partly rescue the collagen-related functional defects in the extracellular matrix [49]. Accordingly, NMD-inhibiting approaches have been considered as a promising treatment for this genetic disease [49,50,51]. Notably, however, Ullrich disease is unrelated to tumorigenesis. Yet this example shows a possibility that similar mechanisms may exist in cancer cells. It will be interesting to investigate whether such partially functional peptides also play a role in tumorigenesis.

In cancer biology, NMD has been reported to be important for cellular adaptations to hostile microenvironments and tumorigenesis [52]. Cellular stresses, such as hypoxia and endoplasmic reticulum stress, can inhibit NMD activities, leading to stabilization of mRNAs and the progression of tumors [53]. One potential link between NMD and tumorigenesis is that NMD is required for the survival of stem and progenitor cells. Weischenfeldt and colleagues reported that in mice, depletion of the core NMD factor UPF2 (UPF2 regulator of nonsense transcripts homolog (yeast)) resulted in significant upregulation of transcripts enriched with processed pseudogenes and alterations in regulated AS events, and rapid and lasting elimination of hematopoietic stem cells [54].

Another NMD regulator, UPF1 (UPF1 regulator of nonsense transcripts homolog (yeast)), has been suggested to be essential for DNA replication and S phase progression in human cells. Unexpectedly, however, this regulatory function of UPF1 has been suggested to be independent of the NMD pathway [55]. Yet Varsally and Brongna inferred from bioinformatics analyses that UPF1 interacted with proteins associated with nuclear RNA degradation and transcription termination. They thus suggested that UPF1 was involved in cellular processes that could indirectly impinge on DNA replication [56]. These observations suggest that although UPF1 participates in the regulations of both NMD and cell proliferation, whether these two biological processes are causally related to each other remains to be determined.

AS-NMD coupled regulation has been reported for the *YT521* (YTH domain containing 1) gene, a ubiquitously expressed splicing factor. In cancer cells, hypoxia shifted the splicing of *YT521* from protein-coding isoforms to non-coding isoforms, which were then targeted by NMD for degradation [57]. The resulting changes in the expression level of *YT521* influenced the splicing of such cancer-associated genes as *BRCA2* (breast cancer 2) and *PGR* (progesterone receptor) [57]. In fact, the splicing pattern of *YT521* has been suggested to be a prognostic factor of endometrial cancer [58].

The widely studied apoptosis regulator caspase-2 has also been reported to be conditionally regulated by AS-NMD [59,60]. In most tissues, the pro-apoptotic isoform Caspase-2L is predominant. The short isoform Caspase-2S shows anti-apoptotic activities [59], and has been found to be upregulated in cancer cells [61]. The primary transcript of the caspase-2 gene includes 12 exons. Exon 9 is specifically inserted into Caspase-2S, generating a PTC at the beginning of exon 10 [60]. In fact, the Caspase 2L/2S isoform ratio was found to be over 100 in leukemia cells (U937, KG1), carcinoma cells (HeLa, HCT116, HepG2, HT29), and immortalized cells (293T, Chang) [60]. To investigate whether this isoform bias was related to NMD, Solier and colleagues quantified Caspase 2L and 2S in a spectrum of cancer cell lines after inhibiting protein translation using cycloheximide. They reported that the inhibition of protein translation induced the accumulation of Caspase-2S mRNA without affecting Caspase-2L mRNAs. This result suggested a short half-life of Caspase-2S and the involvement of the NMD mechanism in regulating the Caspase 2L/2S ratio [60]. Together, the above observations support the involvement of AS-NMD in the regulations of apoptosis.

Of note, AS-NMD does not necessarily lead to downregulation of the affected gene. The cell division regulator H-Ras exemplifies this complexity in AS-NMD regulation. An intronic mutation in H-Ras was found to affect the 5' splice site of an exon named IDX, leading to inclusion of IDX and an increased level of H-Ras expression [62,63]. Interestingly, inclusion of IDX introduced a potential PTC [63], which directed the transcript to NMD [64]. Unexpectedly, however, the supposedly short-lived IDX-containing transcript (termed “p19”) was stably expressed in Hela cells [65]. There has been evidence that normally NMD-sensible transcripts can become NMD-resistant under stress conditions such as hypoxia [66,67], which might be the cause of stable expression of p19 in Hela cells. p19 could interact with the scaffolding protein RACK1, which facilitated the assembly of protein complexes in different signaling pathways [65]. p19 has been reported to regulate the activity of telomerase. The overexpression of p19 could induce the G1/S phase delay, thus maintaining the cell in a reversible quiescence state to avoid apoptosis [68].

In pancreatic adenosquamous carcinoma, somatic mutations frequently occurred in the NMD regulator UPF1. These mutations could result in disruptions of UPF1 splicing and UPF1-regulated NMD activities. The compromised NMD activities could lead to the accumulation of malignant mRNAs. One example is the transcript isoform of p53, alt-PTC-IVS6-p53, which encodes a protein with dominant-negative activities [69].

In breast cancer, RNAi-mediated knockdown of integrin α3β1 in breast cancer cells caused changes in the splicing pattern of cancer-related genes and reduced tumorigenicity [70]. These changes might alter the 3'UTRs or generate PTCs in the affected genes, causing the mRNAs to be targeted by NMD [70,71]. Particularly, the altered splicing pattern of cyclooxygenase-2 (Cox-2) in α3β1-deficient cells was found to yield NMD-sensitive isoforms, which included a retained intron (and a PTC within the intron) and changed 3'UTRs [71]. Of note, the induction of Cox-2 by integrin α3β1 was reported to promote tumor progression. The AS-NMD regulation of Cox-2 was thus proposed to play an important role in the tumorigenesis of breast cancer [71].

## 3. Alternative 5'UTR and Translational Regulations in Cancer

ARS may occur in 5' and 3'UTRs [72]. 5'UTRs contain at least two types of translational regulatory elements—uORFs and G4s. The presence or absence of these regulatory elements can be altered by AS or AP [29,73,74,75]. These two types of regulatory mechanisms may occur separately or concurrently [76]. Both uORFs and G4s can significantly repress the translation of the downstream coding sequences [28,77]. A uORF should contain one AUG codon, one stop codon, and at least one non-stop codon in between. By definition, the AUG codon should be located in 5'UTR, but not necessarily in the first exon. The stop codon can be located either in 5'UTR or in CDS [74]. The sequence contexts of 5'UTRs can influence the efficiency of translational regulations of uORFs, including the length of 5'UTR, the number of uORFs in the 5'UTR, the Kozak sequence context of the uORF, and whether the uORF overlaps with the downstream main CDS [77,78]. uORFs may also induce NMD because these elements by definition include one stop codon, which is usually located upstream of the canonical stop codon [74]. uORFs occur in more than 40% of human transcripts [77,79], and are selectively constrained possibly because of their repressive nature [73]. Indeed, uORFs have been implicated in a number of human diseases [77,80,81].

**Figure 4:**
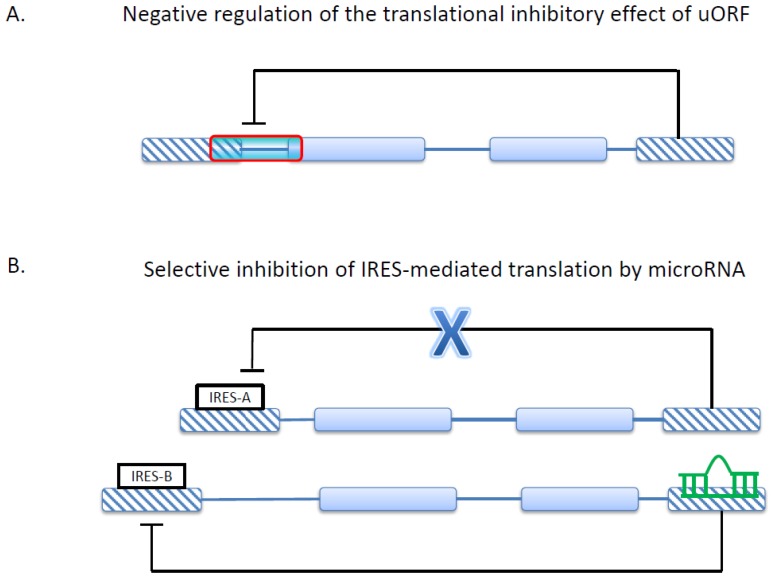
Two types of regulatory interactions between 5'UTR and 3'UTR. (**A**) 3'UTR can negatively regulate the translational inhibitory effect of the uORF in the same transcript; (**B**) By binding to 3'UTR, microRNAs can selectively regulate the activities of IRESs. Note that this illustration does not show the exact transcript structures of the genes (HER-2 or VEGF-A) mentioned in the text.

uORFs have been found in the transcripts of cancer-related genes. For example, the HER-2 (human epidermal growth factor receptor 2) oncogene expresses mRNAs with uORFs, which have been shown to regulate the translation of HER-2 [82]. Unexpectedly, however, the HER-2 mRNA is more efficiently translated in cancer cells than in normal cells despite the presence of uORF in the transcripts in both cell types [83]. This cell type-specific regulation has been reported to rely on 3'UTR, which can counteract the inhibitory effect of uORF in cancer cells [84] (Figure 4A). This is an excellent example of 5'UTR and 3'UTR interacting with each other to regulate gene expression. The prevalence of this 5'–3' interaction remains unclear. Notably, however, the lengths and compositions of both 5' and 3' UTRs can be significantly affected by AS, which positions AS as an upstream regulatory switch.

The regulatory effects of uORFs are well illustrated in the case of the C/EBP (CCAAT/enhancer binding protein) transcription factors (C/EBP α, β, γ, δ, ε, and ζ), which have been reviewed in detail by Wethmar *et al.* [80]. C/EBP α and β, both containing uORF-bearing transcript isoforms, can regulate the proliferation and differentiation of multiple cell types. Importantly, the C/EBP transcription factors have been found to be involved in malignant transformation [85]. The full-length peptides of C/EBP α (termed “α-ext” and “p42”) and β (“LAP*” and “LAP”) contain an *N*-terminal transacting domain and a regulatory domain, which can induce cell differentiation and inhibit proliferation. Meanwhile, the short, N-truncated peptide isoforms (“p30” for C/EBP α and “LIP” for C/EBP β) have repressive effects on C/EBP target genes [86]. Of note, the mRNAs of α-ext and LAP* both contain an out-of-frame uORF, which has been reported to be important for the balances between long and short isoforms of the C/EBP genes [87]. Increased LIP/LAP isoform ratios have been observed in Hodgkin lymphoma, anaplastic large cell lymphoma [88], and aggressive breast cancers [89]. In addition, mutations in the C/EBP α gene in acute myeloid leukemia led to the loss of p42 expression but left the expression of p30 unaffected [90,91], implying the importance of C/EBP isoform balance in tumorigenesis.

Alternative 5'UTRs have been reported to differentially regulate the translation of cancer-related genes. A well-known example is tissue-specific 5'UTR isoforms of BRCA. Sobczak and Krzyzosiak found that longer-5'UTR mRNAs were expressed only in breast cancer tissues, whereas shorter-5'UTR mRNAs were expressed only in normal mammary gland tissues [92]. Importantly, the longer 5'UTR mRNAs were translated at a significantly lower efficiency, yielding a reduced protein expression level [92]. Also in breast cancer, the oncogene Mdm2 was found to be transcribed from alternative promoters, leading to alternative 5'UTR sequences [93]. The short 5'UTR isoform has been shown to be responsible for the elevated protein expression level of Mdm2 in a number of soft tissue tumors [94]. Interestingly, the 5'UTR of Mdm2 has been suggested to confer resistance to rapamycin (an immunosuppressant)-induced translational suppression [95].

One of the most extensively studied types of cancer regulator, the estrogen receptors (ERs), are also subject to 5'UTR-mediated translational regulations. One member of the ER family, ERβ, was found to be transcribed into 5'UTR-isoforms. Furthermore, the translational regulatory effects of the 5'UTRs were subject to carcinogenesis-related modulation [96]. Smith and colleagues reported that 5'UTR isoforms of ERβ were tissue-specifically expressed between normal cells, and differentially expressed between paired tumor and normal tissues in lung and breast cancer. They also demonstrated that uORFs in ERβ significantly repressed protein translation in a variety of cancer cell lines (BT-20, HB2, MCF7, MDA-MB-231, and MDA-MB-453) [96]. Interestingly, the translation of ER genes may also be regulated by G4. Transcription from alternative promoters of ERα (or ESR1) leads to tissue-specific 5'UTR-isoforms, which contain structurally stable G4s. An *in vitro* study demonstrated that one of the G4 structures in ERα possessed strong translational repressive activity [75].

The negative regulator of Wnt signaling pathway and a tumor suppressor gene, *Axin2*, has been reported to include three 5'UTR-isoforms. Although the role of Axin2 as a tumor suppressor remains controversial [97,98], the translation of this gene has been suggested to be regulated in a 5'UTR-dependent manner in tumors. Hughes and Brady showed that both the overall gene expression level and the relative proportions of the three isoforms were altered in lung and colon cancer. Furthermore, the translational efficiencies of the three 5'UTR-isoforms were considerably modulated in the tumor tissues [99].

An additional translational regulatory element in 5'UTR is IRES, a sequence motif for cap-independent translational initiation. IRES has been found in a variety of cancer-related genes. Of note, the presence/absence of an IRES can be regulated either by AS (e.g., XIAP [100] and Apaf-1 [101,102]) or AP (e.g., VEGF-A [103] and FGF1 [104]). Cellular stresses (such as hypoxia) that inhibit cap-dependent translation may elicit IRES-mediated translation [105]. Yet IRESs of different genes respond to stresses differently, even under the same conditions. For example, during apoptosis, the IRES of Apaf-1 (apoptotic peptidase activating factor 1; one of the hubs in the regulatory network of apoptosis) is active, while that of XIAP (X-linked inhibitor of apoptosis) is inhibited [106]. Fibroblast growth factor 1 (FGF1), a critical regulator in cell signaling [107], can be transcribed into four 5'UTR isoforms because of tissue-specific choice of alternative promoters. Two of the 5'UTR isoforms have high and condition-dependent IRES activities *in vivo* [104]. The protein expression level of FGF1 can thus be regulated and coordinated between the transcription and the translation level through alternative 5'UTR selection.

IRES-mediated translation can be further complicated by the involvement of microRNAs (Figure 4B). A good example is the regulation of the angiogenesis factor VEGF-A (vascular epidermal growth factor A), which was nicely reviewed in [105]. VEGF-A is transcribed to at least nine different mRNA isoforms. Two IRES elements resulting from AP were found in the VEGF-A isoforms, termed IRES-A and IRES-B [103]. These two IRES elements give rise to protein isoforms of different lengths. Interestingly, miR-16, a cancer-related regulator, specifically downregulates the IRES-B-mediated translation but not the overall expression or mRNA stability of VEGF-A [108] (Figure 4B). This downregulation of a specific IRES isoform has been found to have an anti-angiogenic effect [105]. Interestingly, the expressions of VEGF-A isoforms have been reported to be negatively regulated by an uORF located within an IRES [103]. This example shows how multiple regulatory mechanisms (ARS, uORF, IRES-mediated translation, and microRNA regulation) may be integrated to determine cellular phenotypes. The interactions between 5'UTR- and 3'UTR-mediated regulations are shown in Figure 4.

Of note, the splicing of VEGF mRNAs appears to be regulated by another intracellular protein, TIA-1 (T-cell intracellular antigen). Hamdollah Zadeh *et al.* [109] reported that an endogenously truncated splicing variant of TIA-1 (sTIA-1) was expressed in colorectal carcinomas but not in adenoma cell lines. They also showed that knockdown of sTIA-1 or overexpression of the full length TIA-1 (flTIA-1) induced the expression of an anti-angiogenic VEGF isoform (VEGF-A_165_b). Interestingly, sTIA-1 could prevent the binding of flTIA-1 to VEGF-A_165_b, thus hampering the flTIA-1-facilitated translation of VEGF-A_165_b [109].

## 4. Alternative 3'UTR and MicroRNA-Mediated Gene Regulation in Cancer

**Figure 5:**
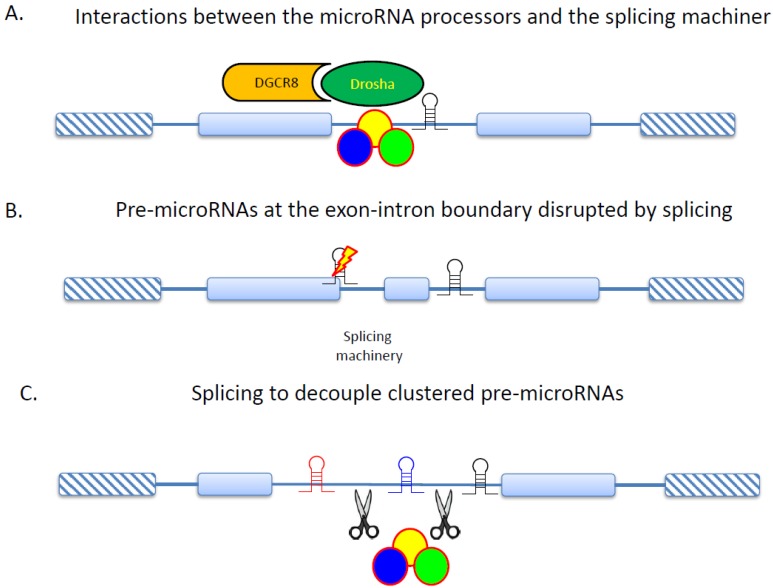
Connections between AS and the biogenesis of intronic microRNAs. (**A**) microRNA processors interact with the splicing machinery and participate in RNA splicing; (**B**) Splicing can disrupt the biogenesis of microRNAs that are located at the exon-intron boundaries; (**C**) Splicing can serve to delineate microRNAs that are clustered in an intron.

MicroRNAs have been demonstrated to contribute significantly to the tumorigenesis of multiple types of cancer [110,111]. These noncoding RNAs are generated from pre-microRNAs by a specific RNA processing machinery that includes Drosha, DGCR8 (DGCR8 microprocessor complex subunit), and other accessory proteins [112]. Most of the pre-microRNAs reside in intergenic regions [113]. Interestingly, however, hundreds of microRNAs have been found to derive from the intronic regions of coding genes [114,115]. The maturation of these intronic microRNAs has been suggested to depend mainly on the splicing machinery instead of the commonly used Drosha/DGCR8 complex [116,117,118,119]. This genic source of microRNA implies correlations between AS and microRNA-mediated gene regulations. Of note, in the case of AS, intronic and exonic regions are interchangeable. The intronic regions that harbor pre-microRNAs may be spliced into coding sequences, thus preventing the biogenesis of these microRNAs. However, this hypothetical AS-microRNA association awaits experimental clarifications (Figure 5).

The molecular connection between AS and microRNA biogenesis has been supported by the finding that Drosha and DGCR8 could be co-precipitated with supraspliceosome [120]. Interestingly, inhibition of RNA splicing could result in the upregulation of microRNAs, while knockdown of Drosha increased the splicing activity [120]. These observations seem to imply that Drosha and the splicing machinery compete with each other for intron substrates (Figure 5A). In fact, DGCR8 has been suggested to participate in AS based on the observation that DGCR8 could alter the ratios of alternative isoforms by cleaving and destabilizing mRNAs that harbored DGCR8 binding sites in their cassette exons [121,122]. In another study, RNA splicing was found to suppress the biogenesis of pre-microRNAs that overlapped the exon/intron boundary in a tissue-specific manner. This suppression nevertheless did not affect pre-microRNAs that fell completely within introns [123]. These observations are understandable because pre-microRNAs that overlap with exon/intron boundaries will be disrupted if these boundaries are disconnected during RNA splicing. Such splicing-caused disruptions do not occur in intronic regions, thus leaving pre-microRNAs in these regions intact (Figure 5B). Still another interesting finding is that Drosha could promote the splicing of an alternative exon in the eIF4H (eukaryotic translation initiation factor 4H) gene in a cleavage-independent manner [124]. This finding, however, appears to contradict the abovementioned hypothesis that Drosha and the splicing machinery compete with each other for intron substrates. The interactions between microRNA processors and the splicing machinery, and the biological functions of such interactions, need to be further clarified.

One possible mechanism of intronic microRNA regulation is self-targeting. Computational analyses indicated that a considerable proportion of intronic microRNAs could target their own host genes [125]. This autoregulation was suggested to decrease the fluctuations in the expression level of the host gene, thus maintaining stable gene expressions [125]. However, this regulatory model has not been experimentally validated. An alternative mechanism is that an intronic microRNA can silence the genes that are functionally antagonistic to its host gene [126]. This mechanism was reported for apoptosis-associated tyrosine kinase (AATK), whose transcription generated the intronic microRNA miR-338. AATK was essential for neuronal differentiation. Interestingly, miR-338 was found to target a group of mRNAs whose protein products negatively regulated neuronal differentiation [126].

The involvement of intronic microRNAs in tumorigenesis has not received wide attention so far. Berillo *et al*. [115] computationally analyzed hundreds of host genes of intronic microRNA whose protein products were implicated in esophageal, gastric, small bowel, colorectal, and breast cancer development. They identified 1751 binding sites of intronic microRNAs in the 5'UTRs, CDSs, and 3'UTRS in 478 mRNAs of these host genes [115]. This study implied a cancer type-specific network of intronic microRNA regulations. Nevertheless, these computational predictions remain to be experimentally verified. Another study focused on intron-21 of focal adhesion kinase (FAK), a gene related to focal adhesion, cell proliferation, and tumorigenesis [127]. This specific intron contains the precursor of miR-151 [128]. The bioinformatically-predicted target genes of miR-151 include regulators of cell cycle (e.g., SCC-112 and Gas2), whose expression is implicated in the development of cancer [129,130]. Another important example is let-7c, an intronic microRNA that serves as a tumor suppressor. The expression of let-7c was reported to be influenced by both the host gene promoter and the intronic promoter upstream of the pre-microRNA [131]. The host gene promoter responded to the anti-cancer drug ATRA (for acute myelogenous leukemia) by adapting a more open chromatin conformation, leading to upregulation of let-7c [131]. However, the epigenetic marks of the intronic promoter did not show significant changes upon ATRA treatment. Meanwhile, in prostate and lung adenocarcinoma, both the host gene promoter and the intronic promoter were functional [131]. These observations exemplify the complexity in the regulation of intronic microRNAs expressions. More studies are required to clarify the influences of the host gene promoter and the intronic promoter on intronic microRNA expression, and the functional consequences thereof.

Of note, the biogenesis of intronic microRNAs can be independent of the transcription of their host genes. A number of studies provided evidence that some of the intronic microRNAs had their own promoters that enabled host gene-independent transcription [132,133,134]. Interestingly, this independence in transcription does not necessarily indicate that splicing is decoupled from the biogenesis of intronic microRNAs. A recent study showed that the intronic region harboring the precursors of three independently transcribed human microRNAs—miR 106b, miR 93, and miR 24-1—could be alternatively spliced. Each of the alternative transcripts contained a single pre-microRNA. It was thus suggested that AS might serve to uncouple the expression of clustered microRNAs [134] (Figure 5C).

In addition to the regulation of microRNA biogenesis, ARS can also affect the regulatory effects of microRNAs by altering their target sites. The target sites of microRNAs have been found to be located in 3'UTRs, 5'UTRs [135], introns [136], and coding exons [137]. The delineation and sequence composition of these genic regions are dependent on the splicing pattern of the target RNA. In other words, changes in splicing patterns can lead to alterations in microRNA-mediated gene silencing [31]. Particularly, the shortened lengths of 3'UTRs and the resulting loss of microRNA targeting sites have been considered as an important feature of oncogenesis [138,139]. The changes in 3'UTR length in cancer cells are usually the combined results of AC and AS, which have been proposed to play an important regulatory role in cancer cells [140]. Unexpectedly, however, in mouse fibroblast cells, alternative 3'UTRs were found to have limited influence on the stability and translational efficiency of mRNAs [141]. This observation, nevertheless, has yet to be confirmed in other cell types and in humans.

Also worth noting in the correlation between splicing and microRNA is that pre-microRNAs, most of which reside in intergenic regions, may include introns. The maturation of such microRNAs thus also depends on correct splicing. Zhang and colleagues demonstrated that in nematodes, intron-containing pre-microRNAs could be efficiently spliced into functional forms [142]. This finding indicates that splicing is required for the maturation of intergenic microRNAs, and suggests possible existence of AS isoforms derived from intron-containing pre-microRNAs. This possibility, nevertheless, has not been investigated.

## 5. RNA Splicing and Protein Interactions in Cancer

Dysregulation of protein–protein interactions (PPIs) plays a critical role in tumorigenesis [119,143]. Indeed, PPI network analyses have been used to unravel the molecular mechanism of carcinogenesis [144], to identify drug targets for cancer therapy [145], and to characterize drug-regulated genes [146]. Of note, alternative exons tend to encode intrinsically disordered protein regions (IDRs), which usually serve as PPI interfaces [147,148]. Furthermore, IDRs can convey structural flexibility, present target sites of post-translational modifications, and harbor motifs for physical interactions. All of these structural/functional features may significantly influence PPIs. Therefore, by altering IDRs, AS can lead to widespread rewiring of the PPI network [149,150]. Interestingly, tissue-specifically regulated exons were found to be enriched in cancer-related genes [150], raising the possibility of splicing-associated PPI network rewiring as a contributor to oncogenesis. Recently, it has been reported that tissue-specific alternative exons could promote rewiring of the PPI network in humans and mice [151]. The functional relevance of such rewiring was exemplified by the regulated interaction between Bin1/Amphiphysin II and the GTPase Dnm2, which was important for efficient endocytosis in neural cells [151]. However, splicing-associated PPI network rewiring has not received much attention in oncological research, despite the appreciation of PPIs and RNA splicing separately as important regulatory mechanisms in tumorigenesis.

## 6. Conclusions

RNA has been increasingly recognized as a key player in determining cellular phenotypes. ARS not only increases the functional diversities of the transcriptome and proteome, but also serves as a multi-way valve that critically regulates information flow across genome, transcriptome, and proteome. Although ARS has been extensively studied, a number of important issues remain unsolved. For example, it remains unclear how epigenetic modifications influence local and global patterns of ARS. How are AS, AP, and AC coordinated? What are the proportions of functionally relevant transcript isoforms in different cell types and different developmental stages? How does the cell select splicing factors out of a repertoire to regulate RNA splicing in response to environmental signals/pressures? Does regulated RNA splicing contribute to dosage balance in duplicate genes or protein complexes? How is RNA splicing co-regulated with transcription? Our lack of knowledge about these fundamental issues indicates underappreciation of this important field. Indeed, some of the studies reviewed here merely provide circumstantial evidence to support the involvements of ARS in tumorigenesis. Although a considerable number of cancer transcriptome studies have been conducted, the research field of ARS-coupled regulations in tumorigenesis is still in its infancy. Significant efforts are required to help clarify the roles of ARS in cell differentiation and cancer cell development.

Importantly, ARS is an integral part of the cellular regulatory network [152]. Since multi-level omics data have become increasingly accessible, it is now feasible to explore the networks of transcript isoforms and the integration of such networks into the multi-omics regulation of cellular functions. These issues are particularly important for understanding the molecular mechanisms of tumorigenesis and drug resistance because of the complex nature of cancer cells. Cancer cells may employ multiple strategies to escape from cell cycle control, immune surveillance, apoptosis, and chemical attack. A systematic view that integrates epigenomic, transcriptomic, proteomic, interactomic, and metabolomic regulations thus can provide a panorama of the regulatory network in cancer cells, enabling identification of alternative pathways/targets for drug development, and facilitating therapeutic interference with cancer cell development. Due to its central position in the regulatory network, ARS undoubtedly will be a pivotal part in this integrative model.
